# Mini Nutritional Assessment May Identify a Dual Pattern of Perturbed Plasma Amino Acids in Patients with Alzheimer’s Disease: A Window to Metabolic and Physical Rehabilitation?

**DOI:** 10.3390/nu12061845

**Published:** 2020-06-21

**Authors:** Roberto Aquilani, Alfredo Costa, Roberto Maestri, Matteo Cotta Ramusino, Antonia Pierobon, Maurizia Dossena, Sebastiano Bruno Solerte, Anna Maria Condino, Valeria Torlaschi, Paola Bini, Mirella Boselli, Mauro Ceroni, Daniela Buonocore, Federica Boschi, Mariella Bruni, Manuela Verri

**Affiliations:** 1 Department of Biology and Biotechnology “Lazzaro Spallanzani”, University of Pavia, 27100 Pavia, Italy; dottore.aquilani@gmail.com (R.A.); maurizia.dossena@unipv.it (M.D.); daniela.buonocore@unipv.it (D.B.); 2 Center of Cognitive and Behavioral Disorders, IRCCS Mondino Foundation, 27100 Pavia, Italy; alfredo.costa@mondino.it (A.C.); matteo.cottaramusino@mondino.it (M.C.R.); paola.bini@mondino.it (P.B.); 3 Department of Brain and Behavior, University of Pavia, 27100 Pavia, Italy; mauro.ceroni@unipv.it; 4 Department of Biomedical Engineering of the Montescano Institute, Istituti Clinici Scientifici Maugeri IRCCS, 27040 Montescano (PV), Italy; roberto.maestri@icsmaugeri.it; 5 Psychology Unit of the Montescano Institute, Istituti Clinici Scientifici Maugeri IRCCS, 27040 Montescano (PV), Italy; antonia.pierobon@icsmaugeri.it (A.P.); valeria.torlaschi@icsmaugeri.it (V.T.); 6 Department of Internal Medicine, University of Pavia, 27100 Pavia, Italy; bruno.solerte@unipv.it; 7 Department of Drug Sciences, University of Pavia, 27100 Pavia, Italy; annamaria.condino@unipv.it (A.M.C.); federica.boschi@unipv.it (F.B.); 8 Neurorehabilitation Unit of the Montescano Institute, Istituti Clinici Scientifici Maugeri IRCCS, 27040 Montescano (PV), Italy; mirella.boselli@icsmaugeri.it; 9 General Neurology Unit, IRCCS Mondino Foundation, 27100 Pavia, Italy; 10 Medical Laboratory Service of the Pavia Institute, Istituti Clinici Scientifici Maugeri IRCCS, 27100 Pavia, Italy; mariella.bruni@icsmaugeri.it

**Keywords:** Alzheimer’s disease, plasma amino acids, nutritional state, cognitive tests

## Abstract

Conflicting results about alterations of plasma amino acid (AA) levels are reported in subjects with Alzheimer’s disease (AD). The current study aimed to provide more homogeneous AA profiles and correlations between AAs and cognitive tests. Venous plasma AAs were measured in 54 fasting patients with AD (37 males, 17 females; 74.63 ± 8.03 yrs; 3.2 ± 1.9 yrs from symptom onset). Seventeen matched subjects without neurodegenerative symptoms (NNDS) served as a control group (C-NNDS). Patients were tested for short-term verbal memory and attention capacity and stratified for nutritional state (Mini Nutritional Assessment, MNA). Compared to C-NNDS, patients exhibited lower plasma levels of aspartic acid and taurine (*p* < 0.0001) and higher 3-methylhistidine (*p* < 0.0001), which were independent of patients’ MNA. In comparison to normonourished AD, the patients at risk of and with malnutrition showed a tendency towards lower ratios of Essential AAs/Total AAs, Branched-chain AAs/Total AAs, and Branched-chain AAs/Essential AAs. Serine and histidine were positively correlated with verbal memory and attention capacity deficits, respectively. Total AAs negatively correlated with attention capacity deficits. Stratifying patients with AD for MNA may identify a dual pattern of altered AAs, one due to AD per se and the other linked to nutritional state. Significant correlations were observed between several AAs and cognitive tests.

## 1. Introduction

Amino acid (AA) levels in the blood and cerebrospinal fluid of patients with Alzheimer’s disease (AD) were measured to detect biochemical signature for early diagnosis, given that AAs, in addition to their nutritional characteristics, are essential for brain neurotransmission [1] and receptor signalling pathways [2]. Although alterations in AA metabolism in AD have been reported to be an early marker of neurodegeneration [3,4], studies have shown inconsistent results: some have highlighted higher plasma AA levels in AD subjects compared to normal controls, while others have highlighted lower or unchanged levels [5,6]. The levels of plasma AAs were also investigated to understand the overtime changes from healthy conditions to subjective memory complaint, to mild cognitive impairment, to AD [7]. Recently, a study documented that levels of blood branched-chain aminotransferase protein (BCAT), the enzyme that regulates Branched Chain AAs (BCAAs) and glutamate metabolism, and glutamate were higher in AD in comparison to healthy controls. This helped to increase the reliability of neurocognitive assessment in distinguishing healthy subjects and subjects with AD [8].

Epidemiologic studies have found inverse associations between plasma BCAAs and increased risk of all dementias, AD [9], cognitive decline and cerebral atrophy changes [10].

However, in another study, no differences were found in plasma BCAAs among healthy controls, subjects with mild cognitive impairment and subjects with AD [11].

The basic idea behind the current study was that we may be able to achieve more homogenous, reliable plasma AA profile measures by relating plasma AAs to patients’ nutritional and metabolic states. Several factors may influence the levels of circulating AAs in AD including both physiological factors such as protein intake and body proteolysis [12,13] and pathological factors such as inflammation [14], in which oral [15] and intestinal [16] dysbioses may play a role, insulin resistance [17], oxidative stress [18], skeletal muscle mitochondrial dysfunction following β-amyloid (Aβ) deposition [19,20,21], and wasting of lean body mass [22].

The reliability of plasma AAs is of clinical importance because AA alterations may suggest body/muscle wasting. In addition, in the presence of AA alterations, we may suspect a progressive failure of neuronal networks and neurotransmitter systems, which causes both synaptic loss and dysfunction [23]. In fact, several circulating AAs, after crossing the blood-brain barrier (BBB), serve as precursors of neurotransmitters in the brain [1]. The AAs cross the BBB in competition with each other [1].

Therefore, in the current observational, prospective study, we measured plasma AA levels in a cohort of subjects with AD. Then, we related the plasma AA levels to both patients’ nutritional state according to a clinical assessment of nutritional state (Mini Nutritional Assessment, MNA) and metabolic state such as insulin resistance. We hypothesised that the AD subjects may be characterised by two diverse perturbed patterns of plasma AA levels, one due to their primary disease (AD) per se and the other linked to the deterioration of their nutritional state. The second hypothesis we formulated was that plasma AAs may be associated with patients’ cognitive functions.

## 2. Materials and Methods

### 2.1. Population

A total of 71 individuals—17 control subjects and 54 patients with dementia that was possibly due to AD (37 males, 17 females; 74.63 ± 8.03 yrs; 3.2 ± 1.9 yrs from symptom onset, according to information supplied by caregivers)—were included in this study. Patients were consecutive cognitively impaired subjects, who underwent diagnostic workup in the Department of General Neurology at the National Institute of Neurology IRCCS Mondino Foundation, Pavia, Italy, between 2014 and 2018. Etiological diagnosis of dementia was formulated based on in vivo evidence of Alzheimer’s pathology in cerebrospinal fluid (CSF): a biomarker profile was considered suggestive if Aβ_1–42_ < 599 pg/mL, tau > 404 pg/mL, p-tau > 56.5 pg/mL, or Aβ_1–42_/tau < 1.6, Aβ_1-42–_/p-tau < 11.5 [24,25]. Exclusion criteria were the following: history of significant neurological or psychiatric illnesses or presence of other diseases precluding enrolment, visual and auditory acuity that was inadequate for neuropsychological testing, and use of drugs with significant central nervous system anticholinergic activity. All stages of AD were represented, with a marked prevalence for mild and moderate AD [26,27]. At the time of blood sampling and CSF collection, some patients were taking choline supplements but not anticholinesterase drugs. With regard to anticholinesterase drugs, 75.9% of the patients had stopped taking them at least two weeks before blood and CSF sampling, 18.5% did not take them because of bradycardia, and 5.6% had not started taking them yet. Moreover, the recruited AD patients were not on corticosteroid nor hormone treatment.

For control group (C) we selected age-matched subjects without cognitive impairment (no neurodegenerative subjects, NNDS; C-NNDS) who underwent neurological consultation and brain MRI scan for reasons unrelated to cognition (paresthesias, migraine or headache, dizziness, auditory disturbances, and lower limb pain). Subjects suffering from diabetes, heart failure, chronic obstructive pulmonary disease, renal disease or endocrinopathies, or involved in discretional physical activity were excluded because all these factors may be responsible for changes in the plasma AA profile. There were ten females and seven males; mean age was 71.80 ± 6.30 yrs; body mass index (BMI) was 25.7 ± 4.9 kg/m^2^ (Table 1). Collection and analysis of blood samples were carried out at the same Neurological Institution as for the AD patients.

The study was conducted in accordance with the Declaration of Helsinki, and the protocol was approved by the Ethics Committee of Ospedale San Raffaele-Istituto di Ricovero e Cura a Carattere Scientifico-Milano, Italy (Project identification code: MAIR2016; ethical approval: 130/INT/2016; date: 8 September 2016). All subjects or their legal representatives gave their informed consent for inclusion before they participated in the study. Participants did not receive financial compensation.

### 2.2. Procedures

#### 2.2.1. Clinical Evaluations

Enrolled patients all underwent complete clinical, neurological, and neuropsychological assessment and brain magnetic resonance imaging (MRI).

In addition to the physical examination, routine blood tests, and anthropometric measurements (weight, kg; height, cm; body mass index, BMI, kg/m^2^; mid-arm circumference, cm), clinical evaluation included an assessment of patients’ nutritional state by means of a Mini Nutritional Assessment (MNA) score [28]. A score below 17 denotes a state of malnutrition, a score between 17 and 24 denotes risk of malnutrition, and a score above 24 identifies a normal nutritional state.

#### 2.2.2. Neuropsychological Assessment

The formal neuropsychological examination included a Mini Mental State Examination (MMSE: a score < 24 denotes cognitive impairment) and Attentive Matrices. Thus, Digit Span (Digit Span < 3.75) [29], which assesses short-term verbal memory, Trail Making Test [30,31] TMT-A (visuospatial selective attention), and Trail Making Test TMT-B (divided attention) were evaluated. Time needed (in seconds) to complete Part A is used as a measure of psychomotor speed (TMT-A < 93″), and time needed to complete Part B is used as a measure of alternation/flexibility, inhibition/interference control, mental tracking, and attentional set-shifting (TMT-B < 282″).

#### 2.2.3. AD Biomarker Measurements in CSF

CSF samples were centrifuged for 10 min at 1800× *g* at 4 °C within 3 h of collection. The samples were then divided into aliquots of 0.5 mL in polypropylene tubes and stored at −80 °C.

Measurement of CSF β-amyloid, tau, and p-tau was performed using standardised ELISA (Fujirebio Europe, Ghent, Belgium).

#### 2.2.4. Plasma AA Measurements

In both patients and C-NNDS, at 8 a.m. after 12 h of overnight fasting, blood samples were drawn from an antecubital vein and immediately delivered to the laboratory where plasma was obtained from heparinised blood using centrifugation (800× *g*, 15 min).

The concentration of free amino acids in the plasma was measured using an AminoQuant II amino acid analyser, based on the HP 1090 HPLC system, with fully automated precolumn derivatisation. Both orthopthalaldehyde (OPA) and 9-fluorenyl-methyl-chloroformate (FMOC) reaction chemistries were used, according to the manufacturer’s protocol. Measurements were made by injecting 1 μL of the derivatised mixture and measuring absorbance simultaneously at 338 and 262 nm [32]. Plasma concentrations were expressed as μmol/L. The measurements of the plasma amino acids were carried out in triplicate by the same laboratory. The mean of the three measurements was calculated and adopted. The characteristics of the method were based both on precision and standardisation properties (unpublished data): precision, relative standard deviation (RSD) was 1.13%; reliability (bias) was 10.37%; the lower limit of quantitation was 0.18 ng/mL; the limit of detection was 0.6 ng/mL. For the measurements in triplicate, the intra-day variability (RSD) was 3.21% and the intervariability was 4.77%.

The AAs measured were: (1) Essential AAs (EAAs): leucine, valine, isoleucine, lysine, threonine, phenylalanine, methionine, and tryptophan. The first three EAAs constitute the Branched-chain AAs (BCAAs); (2) non-EAAs: aspartic acid, glutamic acid, histidine, asparagine, serine, glutamine, 3-methyl-histidine, arginine, citrulline, glycine, alanine, taurine, and tyrosine.

As AA levels in venous plasma mainly reflect the metabolism of the tissue/organ where the blood is drawn from, we discussed AAs mainly in relation to skeletal muscle tissue.

#### 2.2.5. Insulin and HOMA-IR

At 8 a.m., peripheral venous blood samples were drawn for assay of insulin (µU/mL) levels (commercial kits Cord-CT Radioimmunoassay Kit CIS France, Coat A Count Insulin, D.P.C., Los Angeles, CA, USA). Insulin resistance was calculated by the HOmeostasis Model Assessment (HOMA; normal value < 2.4) [33,34].

### 2.3. Statistical Analysis

Descriptive statistics are reported as mean ± SD for continuous data and as a number (percentage frequency) for discrete variables. The Shapiro–Wilk test, supported by visual inspection, was used to assess the normality of the distribution of continuous variables. Since several variables violated the normality assumption, non-parametric statistics were preferred. Accordingly, between group comparisons were carried out by the Kruskal–Wallis test. When three or more groups were compared, if a global significant difference was observed, post-hoc paired comparisons were carried out (Dunn–Sidak test).

The association between neurocognitive tests and plasma AAs, between plasma AAs and CSF concentrations of AD biomarkers, between neurocognitive tests and nutritional state (MNA) and insulin resistance (HOMA-IR) and blood glucose were assessed by Spearman’s rank correlation coefficient.

All statistical tests were two-tailed and statistical significance was set at *p* < 0.05. When appropriate, false discovery rate was controlled at 5% using the Benjamini–Hochberg method. All analyses were carried out using the SAS/STAT statistical package, release 9.4 (SAS Institute Inc., Cary, NC, USA).

## 3. Results

### 3.1. Patients General Characteristics

The study showed that AD patients (Table 1) had normal anthropometric measures (body weight; body mass index, BMI; mid-arm circumference [35]) and biohumoral variables, with a slight increase in the erythrosedimentation rate. These variables were not significantly different from those found in C-NNDS. AD patients presented with a state of insulin-resistance (HOMA-IR). Notwithstanding insulin resistance, the patients had normal glycosylated haemoglobin. Circulating proteins were mildly lower than normal values but albumin levels were still normal.

According to MNA, 51.9% patients (28/54) had a normal nutritional state, 33.3% (18/54) were at risk of malnutrition, and 14.8% (8/54) were malnourished.

### 3.2. Plasma AA Concentrations in AD Patients and in C-NNDS

With regard to plasma AA concentrations, in comparison to C-NNDS, AD patients had 45% (9/20) of altered individual AAs: 35% were lower (aspartic acid, glutamic acid, histidine, asparagine, serine citrulline, taurine; *p* < 0.02), 10% were higher (3-methyl-histidine, 3-MHI, lysine; *p* < 0.001). Moreover, the AD patients exhibited a lower content of Total AAs (TAAs; *p* < 0.035), a trend towards higher EAA/TAA ratio (*p* = 0.057), and a lower ratio of BCAAs/EAAs (*p* = 0.004).

The distributions of the altered AAs in AD patients stratified for nutritional status were similar in the three subgroups: 25% in both normonourished and at risk of malnutrition patients, 20% in malnourished patients.

To investigate whether the AA alterations were attributable to the primary disease per se and not to altered nutrition, we compared C-NNDS with each MNA-stratified subgroup (Table 2) and observed that each patient subgroup, in comparison to C-NNDS, shared the same pattern of significantly altered AAs: lower aspartic acid and taurine, and higher 3-MHI. This indicated a pattern of perturbed AAs independent of patients’ nutritional state.

To better understand the contribution of the nutritional state to the altered circulating AAs, these substrates were analysed in the 3 patient subgroups. Compared to normonourished patients, the subjects at risk of malnutrition had lower levels of both BCAA/TAA (*p* = 0.038) and BCAA/EAA (*p* = 0.026) ratios, whereas malnourished patients displayed a lower BCAA/EAA (*p* = 0.035) ratio. No differences were found between patients at risk of malnutrition and malnourished patients. After pooling together these two subgroups (combined group: CG), CG compared to the normonourished subgroup (Table 3) showed lower levels of leucine (*p* = 0.047), valine (*p* = 0.047), and tended to have lower BCAA levels (*p* = 0.06) and lower EAA/TAA− (*p* = 0.023), BCAA/TAA− (*p* = 0.009) and BCAA/EAA (*p* = 0.002) ratios.

Of interest, regarding the non-amino acid variables, after their stratification for patient MNA groups (Table 4), CG group (malnourished-MNA Group 1 + at risk of malnutrition-MNA Group 2) in comparison to MNA Group 3 (normonourished AD patients) had significant reductions in body weight, red blood cell count, and haemoglobin concentration.

Thus, we found that HOMA-IR was similar in the various subgroups.

### 3.3. Correlations between Plasma AAs and CSF Concentrations of AD Biomarkers

The study showed several significant correlations between plasma AAs and CSF concentrations of AD biomarkers.

Aβ was negatively associated with histidine (*r* = −0.33, *p* < 0.05). p-tau was positively correlated with serine (*r* = +0.40, *p* < 0.05) and especially glycine (*r* = +0.63, *p* < 0.001). Tau was positively linked to aspartate (*r* = +0.33, *p* < 0.05), serine (*r* = +0.39, *p* < 0.05), alanine (*r* = +0.45, *p* < 0.01) and glycine (*r* = +0.55, *p* < 0.001).

To sum up, the results showed that: (1) significant alterations in plasma levels of aspartic acid, taurine, and 3-MHI were present in all patients’ nutritional categories, indicating that they were constitutive of the primary disease; (2) alterations in BCAAs/TAAs, EAAs/TAAs, and BCAAs/EAAs seem to be associated with patients’ nutritional state, showing a tendency to be lower in the CG group than in normonourished AD patients.

### 3.4. Correlations between Non-Amino Acid Variables and Respectively Plasma AAs with Neurocognitive Tests

The study found that the neurocognitive tests were similar in the 3 patient subgroups and showed no significant correlation with nutritional state (MNA) *(r* between MNA and MMSE = +0.20, Digit Span *r* = −0.18, TMT-A *r* = −0.02, TMT-B *r* = −0.25).

Cognitive impairment (MMSE < 24) was observed in 78% of the patients (80, 91, and 71%, in MNA Group 1, 2, and 3, respectively), Digit Span exceeded NV value in 38% of the patients (60, 27 and 38% in MNA Group 1, 2, and 3, respectively), TMT-A exceeded NV value in 43% of the patients (40%, 36%, and 46% in MNA Group 1, 2, and 3, respectively) and TMT-B exceeded NV value in 8% of the patients (20%, 9%, and 4% in MNA Group 1, 2, and 3, respectively).

Moreover, no significant associations were found between neurocognitive tests and insulin resistance (HOMA-IR) (*r* HOMA-IR vs. MMSE = −0.02, Digit Span *r* = +0.08, TMT-A *r* = +0.10, TMT-B *r* = +0.23) nor between neurocognitive tests and blood glucose (*r* blood glucose vs. MMSE = +0.15, Digit Span *r* = −0.21, TMT-A *r* = −0.09, TMT-B *r* = −0.23).

Furthermore, plasma tryglycerides and body weight were negatively correlated with short-term verbal memory (Digit Span; *r* = −0.43 and respectively *r* = −0.47, *p* < 0.05). Serum transferrin was negatively associated with TMT-B (*r* = −0.68, *p* < 0.05). Both serum vitamin B12 and total proteins concentrations were negatively associated (*r* = −0.47 and respectively *r* = −0.49, *p* < 0.05 for both) with TMT-A.

With respect to plasma AAs and neurocognitive tests, serine levels were positively associated with Digit Span (*r* = +0.34, *p* < 0.05). TAAs were negatively associated with both TMT-A (*r* = −0.43, *p* < 0.05) and TMT-B (*r* = −0.55, *p* < 0.05). Histidine levels were positively correlated with TMT-A (*r* = +0.44, *p* < 0.05) and with TMT-B (*r* = +0.82, *p* < 0.01).

No significant correlations between the other AAs and neurocognitive tests were observed.

## 4. Discussion

The results of the study are in line with our initial hypothesis that AD patients may have two different patterns of altered circulating AAs, one due to the primary disease per se, the other likely associated with nutritional conditions. Moreover, the study shows that some circulating AAs correlate with neurocognitive tests such as short-term verbal memory and attention deficits.

The combination of low levels of both aspartate and taurine with higher 3-MHI seems to be constitutive of the AD per se whereas a higher EAA/TAA ratio, a lower BCAA/EAA ratio, and a trend towards lower BCAA levels seem linked to the deterioration of nutritional state.

This research suggests that taking into account patients’ nutritional states may be useful to differentiate the alterations of AAs due to AD from those linked to patients’ nutritional states. Thus, we will only discuss the AA alterations due to AD per se and those linked to patients’ nutritional state.

### 4.1. AA Alterations Linked to AD Per Se

The combination of the two patterns might reconcile the discrepancies in plasma AAs found in previous studies [5,36]. On the other hand, discrepant plasma AA profiles in previous studies should not be surprising because the metabolic conditions of AD patients may be greatly heterogeneous as they are dependent not only on biochemical alterations due to Aβ deposition [37] but also on patients’ protein intake, body muscle proteolysis [38], insulin resistance [17], and severe muscle and brain metabolic derangements [37]. In addition, the overtime changes of these factors can further modify plasma AA contents and profiles. The interplay of all these factors may alter patients’ nutritional state.

In the current investigation, altered AA levels due to AD per se may reflect intracellular metabolic disturbances following Aβ deposition such as (1) difficulties in muscle energy formation leading to increased consumption of aspartic acid, a substrate that plays a key role both in anaerobic and aerobic pathways [37]; (2) muscle hypercatabolism (3-MHI); (3) increased consumption of antioxidant systems leading to low taurine [37]. Muscle hypercatabolism leads to increased AA release that may explain the higher EAA/TAA ratio in AD.

Aspartic acid plays a major role in the production of high energy phosphate compounds (ATP) as it forms the malate-aspartate cycle, which is not only the main mechanism for the oxidation of glucose and lactate [39], but it also regulates the entry of carbon skeletons from AAs into the tricarboxylic chain acid (TCA) cycle [40]. We postulate that in the study population, low plasma aspartic acid levels may be due to unbalanced high ratio cell consumption/diet provision + endogenous formation.

It is noteworthy that higher lysine concentrations in the overall AD population may confirm both the difficulty in muscle aerobic energy formation [5] and impaired protein synthesis [22]. Indeed, lysine, an essential AA, is only used for protein synthesis and is a precursor of carnitine formation, a substance essential for fat acid entry into mitochondria to be oxidised.

Low taurine may be caused by low availability of its precursors, methionine and cysteine. In this study, however, this was not the case, as the levels of taurine precursors were normal. Both inflammation and oxidative stress are likely to explain the low taurine [41,42]. As taurine has antioxidant properties, its low availability might aggravate oxidative stress and intracellular and mitochondrial overload [43].

Elevated 3-MHI reflects hypercatabolism of muscle contractile proteins [44]. Therefore, alterations in plasma AAs linked to the primary disease per se suggest that patients with AD, even if normonourished, have an increased risk of or are developing muscle wasting due to energy deficits and myofibrillar protein catabolism [37].

### 4.2. Correlations between Plasma AAs and CSF Concentrations of AD Biomarkers

The current study cannot explain the associations found between several plasma AAs and the CSF concentrations of the histological hallmarks of AD. We can only formulate some hypotheses.

Future studies targeting contemporary determinations of peripheral and CSF AA concentrations might help explain the mechanisms of these associations.

An excess of plasma histidine, an aromatic AA, may further reduce the BCAA/aromatic AA ratio at the BBB and may limit the BCAA entry to the brain. Reduced brain BCAA availability might reduce the reuptake of interstitial excitotoxic glutamate [45], favouring, in turn, neurodegeneration, increased Aβ formation leading to imbalanced ratio between production and clearance of the Aβ peptide [46].

The important correlation of glycine and serine (which can be converted to glycine) with p-tau protein could be explained by glycine (and glutamate) activation of NMDA receptors [47,48,49]. In AD, NMDA receptors are continuously activated [50].

The link between plasma aspartate and p-tau could be due to aspartate stimulation of the NMDA receptors with subsequent neurotoxicity. Of note, plasma aspartate and glycine are physiologically restricted in their entry to the brain.

Alanine is produced by the BCAA leucine metabolism. The positive correlation between plasma alanine and CSF tau levels could be due to both a possible diversion of leucine from the glutamate/glutamine cycle and to the reduced astrocyte uptake of interstitial excitotoxic glutamate, which is an important factor for neurodegeneration.

### 4.3. Neurocognitive Tests and Patients’ General Characteristics

The study suggests that in AD patients, short-term verbal memory is associated with increases in both tryglyceride levels and body weight. Even though correlation does not necessarily mean a cause-effect relationship, the results are supported by at least two studies, one documenting that short term consumption of a high fat diet by aged animals leads to neuroinflammation and memory deficits [51], the other highlighting that obesity causes impairments of declarative memory [52].

Attention capacity deficits are lower with increased serum levels of vitamin B12, total proteins, and transferrin. Results of both clinical and observational studies reported that vitamin B12 plays an important role in cognition [53]. Given that vitamin B12 is connected with mitochondrial function, myelin integrity, and oxidative stress (and peripheral nerve function) [54], the lack of the vitamin may damage the brain structures deputed to cognitive functions. The inverse relationships between attention capacity deficits, on one hand, and circulating total proteins, transferrin, on the other hand, may account for the beneficial impact of the body/brain protein and AA metabolism and the transferrin modulation of iron transport to the brain. In fact, transferrin is a complex protein system that chelates iron. This metal is involved in brain function and dopaminergic activity. Increasing dopamine signalling, for example, by methylphenidate, is the first-line stimulant treatment for attention deficit/hyperactivity disorder [55]. It is interesting to note that in the study patients, the negative correlations of transferrin with attention capacity deficits seem to be in contrast to the role played by transferrin in mediating the effect of iron on the production and the deposition of Aβ, thus contributing to the pathogenesis of AD [56].

### 4.4. AA Alterations Linked to Nutritional State

With respect to plasma AAs linked to patients’ nutritional state, the patients at risk for malnutrition and those with malnutrition as a combined group (CG), when compared to normonourished AD patients, had a tendency towards lower ratios of EAAs/TAAs, BCAAs/TAAs, and BCAAs/EAAs. It is of note that these AA differences occurred notwithstanding the non-amino acid variable differences between CG and normonourished AD patients. The differences in AA ratios indicate an overconsumption of EAAs, in particular BCAAs, which is not adequately compensated for by diet EAA intakes. Likely, BCAAs were mainly used to produce energy within skeletal muscles, as these AAs represent the preferential substrates for energy production. This is supported by a low BCAA/EAA ratio. Had BCAAs mainly been used for protein synthesis, we would have observed a more balanced BCAA/EAA ratio [18].

Besides their nutritional activities, BCAAs play a crucial role for brain function. These AAs are easily taken up by the brain [57] crossing the BBB in competition for the same transporter with the aromatic AAs phenylalanine, tyrosine and tryptophan [1]. In brain tissue, BCAAs are more catabolised than incorporated into proteins [58]. The first step in the catabolism of BCAAs is the reversible transamination with α-ketoglutarate to produce the neurotransmitter glutamate and branched-chain α-ketoacids (BCKAs). The reaction is catalysed by the branched-chain aminotransferase isozymes (BCAT), cytosolic BCATc [59], and mitochondrial BCATm [60]. In the brain, BCATc explains 70% of total BCAT activity [61]. In the brain, BCAAs provide amino groups of the synthesis of glutamate [62], and BCATc takes part in the glutamate/glutamine cycle [63]. A derivative of BCAA-formed excitatory glutamate is the inhibitory γ-aminobutyric acid (GABA).

Importantly, BCATc is present in neurons that use a variety of neurotransmitters such as cerebral cortex, white matter, internal capsule, globus pallidus, diencephalon, hypothalamus, tuberomammillary nucleus, many midbrain and brainstem nuclei, and spinal cord (grey matter) [45].

A recent research study has documented that blood BCAT proteins were increased in patients with AD in comparison to healthy controls [8]. Blood BCAT proteins and glutamate increase the sensitivity and specificity of Montreal Cognitive Assessment in correctly discriminating healthy subjects and subjects with AD, which is useful for an early diagnosis of AD [8]. The increase in blood BCAT paralleled the increased levels of BCATs found previously in the hippocampus, frontal and temporal cortex, in a post-mortem analysis of AD patients [64]. In this study, BCATm correlated with disease severity (Braak stage).

The importance of BCAAs has been documented in healthy subjects, in clinical and rehabilitation settings [8]. Supplementation of BCAAs improved cognitive performance in healthy subjects [65,66,67,68].

In the clinical setting, supplementation of BCAAs improved cognitive functions in patients with cirrhosis and chronic hepatic encephalopathy [69], in subjects with phenylketonuria [70]. In the rehabilitation ward, intravenous infusion of BCAAs enhanced the cognitive recovery of patients with severe traumatic brain injury [71] and improved recovery from a vegetative or minimally conscious state in patients following traumatic brain injury [72]. BCAA-induced cognitive recovery was subsequently documented in both an experimental model of traumatic brain injury [73] and in humans [74,75].

EAA consumption, when not adequately exogenously compensated, reduces metabolic support not only to muscles but also to vital organs including the brain and the heart [32]. EAA consumption may explain the associations between dementia and nutritional factors, such as the relationships between weight loss and dementia severity and faster progression [22], creatinine and dementia [9], and reduced BMI and medial temporal lobe atrophy [22]. Weight loss has been found to precede cognitive symptoms in AD [76]. We postulate that the higher EAA/TAA ratio that was found in the AD population compared to C-NNDS and the lower EAA/TAA ratio that was found in the AD CG group compared to normonourished AD may reflect two distinct but linked metabolic processes, the former an increase in muscle EAA release, the latter a body EAA overconsumption and/or reduced muscle mass, hence protein/AA content. If this hypothesis is correct, normonourished AD would have an excess of muscle EAA release, whereas non-normonourished AD may have EAA body overconsumption and/or reduced muscle mass.

In AD patients, reduced brain availability in EAAs and BCAAs may aggravate brain energy deficit [77], chemical neurotransmission, synaptic loss and synaptic malfunction [23], and glutamate neurotoxicity [45].

Recent research [9] has documented, in a large cohort of subjects, an association between low levels of BCAAs and increased risk of dementia, independently of conventional risk factors.

In the current study, the presence of perturbed EAA and BCAA levels in AD subjects who are at risk of malnutrition or malnourished might contribute to explaining the association between a lower MNA score and progression to dementia [76].

In the present investigation, the influence of circulating AAs on brain functioning may be witnessed by the associations of some AAs with neurocognitive tests. In a previous study, plasma glutamate correlated with visuo-spatial deficits [2]. However, we found a positive correlation between plasma serine levels and short-term verbal memory, and negative correlations between TAAs and attention deficits. Plasma histidine was positively correlated with attention deficits.

Circulating serine in the brain can be transferred to the stereoisomer D-serine, a potent activator of NMDA glutamate receptor, involved in the formation of new synapses which are important for learning and memory [78].

Notably, patients’ attention capacity is correlated with TAAs and not with a single AA, which suggests the importance for brain function of optimal availability in all the AAs. It is more difficult to explain the positive relationship between plasma histidine and attention deficits, given that it has been reported that subjects affected by attention deficit disorder have significantly lower levels of histidine [79]. As histidine is an aromatic AA, an excess of this AA may limit the entry of phenylalanine and tyrosine into the brain, thus negatively influencing dopaminergic neurotransmission that plays a major role in attention capacity [80].

In the present study, the importance of circulating AAs for brain functioning is also witnessed by the absence of significance of the correlations between neurocognitive tests and HOMA-IR, neurocognitive tests and nutritional state (MNA), and neurocognitive tests and blood glucose. This means that at the disease stage in the study patients, the brain may be more susceptible to AAs than to glucose.

Interestingly, in the study, the AAs which correlated with the cognitive tests were those linked to patients’ nutritional state and not to AD per se, suggesting that diet manipulation or appropriate AA supplementation may benefit AD patients with attentive dysfunction.

## 5. Strengths and Limitations of the Study

The strength of the current study is that administering a simple, clinical nutritional assessment test to AD patients may help physicians to identify patients’ needs for essential and non-essential AAs. Moreover, the study reveals that low plasma levels of aspartate and taurine together with high plasma levels of 3-MHI seem to be constitutive of AD per se.

This study has several limitations that may be overcome by performing well-planned further investigations.

The findings of the study should be confirmed in a larger cohort of patients enrolled in different AD settings. The confirmation of altered AAs in AD including aspartic acid, 3-MHI, taurine, and serine is important for clinical practice because altered AAs can be exogenously manipulated.

Patients’ nutritional state would have been better defined if we had measured body composition. For example, possible impairment of skeletal muscle mass would have allowed us to better understand the mechanisms underlying EAA/TAA ratio. However, the adoption of a simple nutritional test (MNA) may prove useful in a clinical setting to suggest aberrant plasma AA profiles before the occurrence of changes in standard nutrition variables (body weight, albumin levels) [76].

Measurements of AA levels in arterial blood would have allowed us to calculate the AA artero-venous differences and the net muscle release/uptake/no changes of AAs, thus providing more information about skeletal muscle protein/AA metabolism.

The determination of AAs both in plasma and CSF would have allowed us to better understand the possible utilisation of AAs in brain structures.

Patients were not requested to keep a food diary when at home. Analysing patients’ nutritional intakes, mainly the amount and quality of ingested proteins, would have allowed us to have a better understanding of the interplay between AA intake and body/muscle protein metabolism.

Future studies will describe the relationships between plasma AAs and cognitive tests that have not been discussed in this research.

## 6. Suggestions for Clinical Practice and Future Directions

This study provides some useful suggestions for both clinical practice and future investigations, addressing the effects on AD patients of the corrections of the aberrant AA profiles.

- In addition to altered AAs due to AD per se, subjects classified as at risk of or with malnutrition may be at high risk for overtime deterioration of EAAs, especially BCAAs. In these subjects, it would be prudent to monitor the adequacy of nutritional intake, in particular the amounts of both protein quantity and quality. In case of insufficient protein intake, the patients should be supplied with free EAAs/proteins.
- Future studies are needed to explore whether the recovery of aberrant plasma AAs could be relevant to AD subjects not only to maintain or improve the nutritional state but also to positively impact on neurocognition. We hypothesised that for these aims, combined supplemented proteins/AAs and exercise training might improve skeletal muscle mass, the main body tissue of AA store, circulating AAs, and brain availability.

## 7. Conclusions

The study shows that AD patients may be characterised by two altered patterns of plasma AA levels, one due to AD per se and the other likely associated with patients’ nutritional state. Furthermore, the study found the existence of correlations between some AA levels and verbal memory and attention deficits.

## Figures and Tables

**Table 1:** Non-amino acid variables of the entire population of patients with Alzheimer’s disease (AD) and of the control group (C-NNDS).

Variable	AD Patients (*N* = 54)	C-NNDS (*N* = 17)
**Demographic variables**		
Age (years)	74.63 ± 8.03	71.8 ± 6.3
Male gender (%)	37 (69%)	7 (41.2%)
**Anthropometric variables**		
Education (years)	7.6 ± 0.1	9.8 ± 5.3
Body weight (kg)	63.69 ± 12.67	69.1 ± 8.5
Height (cm)	161.16 ± 9.75	164 ± 6.4
Body mass index (kg/m^2^)	24.66 ± 4.71	25.7 ± 4.9
Mid-arm circumference (cm)	26.01 ± 2.83	27.4 ± 3.5
**Biohumoral variables**		
Glucose (NV 70–115 mg/dL)	89.17 ± 15.25	95.2 ± 10.4
Insulin (NV 4–24 µU/mL)	12.63 ± 5.32	-
HOMA-IR (NV < 2.4)	3.84 ± 2.09	-
Glycosylated haemoglobin (4.8–5.9%)	5.59 ± 0.71	5.3 ± 0.9
Total cholesterol (NV < 200 mg/dL)	185.98 ± 32.89	200.3 ± 41.5
HDL cholesterol (NV: M > 55 mg/dL; F > 65 mg/dL)	55.75 ± 16.34	-
LDL cholesterol (NV < 100 mg/dL)	109.49 ± 27.86	-
Transferrin (NV 200–360 mg/dL)	230.29 ± 37.69	-
Iron (NV 59–158 µg/dL)	84.27 ± 25.67	75.6 ± 19.5
Tryglycerides (NV 0–200 mg/dL)	101.51 ± 39.32	132.8 ± 25.6
Vitamin B12 (NV 191–663 pg/mL)	321.50 ± 147.19	352.7 ± 100.5
Folate (NV 3.1–17.5 ng/mL)	6.49 ± 3.11	5.3 ± 2.5
Creatinine (NV: M 0.73–1.18 mg/dL; F 0.55–1.02 mg/dL)	0.90 ± 0.22	0.95 ± 0.16
Total proteins (NV 6.6–8.7 g/dL)	6.29 ± 0.40	6.9 ± 0.7
Albumin (NV 3500–5200 mg/dL)	3696 ± 317	3802 ± 358
White blood cell count (NV 4.00–10.00 × 10^3^/µL)	6.34 ± 1.48	5.8 ± 1.02
Red blood cell count (NV: M 4.30–5.70 × 10^6^/µL; F 3.80–5.20 × 10^6^/µL)	4.34 ± 0.45	4.5 ± 0.6
Haemoglobin (NV: M 13.2–17.3 g/dL; F 11.7–15.5 g/dL)	13.14 ± 1.24	13.8 ± 1.7
Erythrosedimentation rate (NV < 15 mm/1st h)	17.44 ± 17.81	15 ± 6.5
**AD biomarker concentrations in CSF**		
tau (NV < 404 pg/mL)	453.89 ± 408.61	-
p-tau (NV < 56.5 pg/mL)	74.91 ± 35.76	-
β-amyloid (NV > 599 pg/mL)	555.33 ± 359.23	-
β-amyloid/tau (NV > 1.6)	2.22 ± 2.73	-
β-amyloid/p-tau (NV > 11.5)	9.35 ± 8.27	-
**Neurocognitive tests**		
Mini Mental State Examination (MMSE < 24 denotes cognitive impairment)	18.42 ± 7.10	-
Digit Span (NV < 3.75)	3.90 ± 0.85	-
Trail Making Test TMT-A (NV < 93″)	156.33 ± 57.29	-
Trail Making Test TMT-B (NV < 282″)	194.27 ± 107.49	-

Data are expressed as mean ± standard deviation except for gender, expressed as *N* (%). Abbreviations: NV, normal values; HOMA-IR, HOmeostasis Model Assessment-Insulin Resistance; CSF, cerebrospinal fluid.

**Table 2:** Differences in plasma amino acid levels (µmol/L) in control subjects (C-NNDS), malnourished patients with Alzheimer’s disease (AD) (MNA Group 1), AD patients at risk of malnutrition (MNA Group 2), and normonourished AD patients (MNA Group 3). The table shows that the 3-nutritionally stratified AD subgroups, compared to C-NNDS, had 3 altered AAs in common: aspartic acid, taurine, and 3-methyl-histidine.

Amino Acids	C-NNDS (*N* = 17)	AD Patients MNA Group 1 (*N* = 8)	AD Patients MNA Group 2 (*N* = 18)	AD Patients MNA Group 3 (*N* = 28)	*p* Value
Aspartic acid	27.86 ± 4.34	8.88 ± 5.59 ^†^	7.17 ± 2.79 ^‡^	8.86 ± 6.80 ^‡^	<0.0001
Glutamic acid	107.74 ± 52.29	64.63 ± 19.18	60.33 ± 17.32 ^†^	68.68 ± 17.70	0.008
Histidine	60.22 ± 35.79	69.63 ± 8.93	67.17 ± 13.29	71.39 ± 14.14 ^^^	0.025
Asparagine	50.99 ± 14.84	40.25 ± 4.30	39.33 ± 6.54 ^^^	42.71 ± 6.51	0.015
Serine	130.79 ± 27.39	102.13 ± 19.75	96.56 ± 22.57 ^†^	110.00 ± 48.16 ^†^	0.0009
Glutamine	488.95 ± 75.61	515.00 ± 82.56	499.72 ± 83.32	511.07 ± 82.65	0.86
3-methyl-histidine	0.87 ± 0.39	3.25 ± 1.97 ^†^	3.66 ± 1.98 ^‡^	3.88 ± 1.30 ^‡^	<0.0001
Arginine	64.40 ± 19.60	71.75 ± 16.44	63.89 ± 24.23	63.79 ± 22.33	0.62
Citrulline	39.22 ± 7.33	35.88 ± 9.80	33.44 ± 8.94	35.32 ± 11.75	0.15
Glycine	224.84 ± 61.68	234.88 ± 38.80	246.11 ± 93.85	232.79 ± 81.78	0.81
Threonine	129.30 ± 42.21	125.88 ± 23.14	116.56 ± 28.67	118.07 ± 28.58	0.80
Alanine	370.89 ± 79.26	321.50 ± 39.71	371.67 ± 91.31	344.14 ± 74.21	0.29
Taurine	106.93 ± 32.71	82.25 ± 71.18 ^†^	71.72 ± 25.77 ^^^	64.39 ± 15.10 ^‡^	<0.0001
Tyrosine	59.21 ± 17.61	53.50 ± 11.95	54.00 ± 13.56	53.21 ± 12.47	0.65
Tryptophan	47.23 ± 12.37	42.13 ± 11.10	44.22 ± 10.80	46.11 ± 8.36	0.47
Phenylalanine	61.32 ± 24.76	59.63 ± 23.54	52.78 ± 11.31	53.11 ± 10.19	0.67
Isoleucine	61.37 ± 14.60	57.38 ± 14.19	54.17 ± 20.55	60.00 ± 14.70	0.45
Leucine	122.37 ± 25.25	105.38 ± 35.32	102.28 ± 30.76	116.79 ± 24.39	0.08
Lysine	162.26 ± 35.02	219.25 ± 59.62	195.17 ± 44.17	205.21 ± 28.65 ^†^	0.003
Valine	246.49 ± 53.63	205.48 ± 68.88	199.44 ± 59.98 ^	227.73 ± 47.55	0.046
TAAs	2581.83 ± 263.18	2428.11 ± 365.33	2387.83 ± 424.43	2446.96 ± 308.65	0.27
BCAAs	430.23 ± 82.36	368.23 ± 117.08	355.89 ± 110.57	404.52 ± 84.87	0.061
EAAs	891.43 ± 137.92	887.98 ± 223.29	835.44 ± 196.77	902.29 ± 145.03	0.45
EAAs/TAAs	0.34 ± 0.03	0.36 ± 0.04	0.35 ± 0.03	0.37 ± 0.02	0.033
BCAAs/TAAs	0.17 ± 0.02	0.15 ± 0.03	0.15 ± 0.03	0.16 ± 0.02	0.036
BCAAs/EAAs	0.48 ± 0.05	0.41 ± 0.04 ^†^	0.42 ± 0.04 ^†^	0.45 ± 0.03	<0.0001

^^^: *p* < 0.05 vs. C-NNDS; ^†^: *p* < 0.01 vs. C-NNDS; ^‡^: *p* < 0.0001 vs. C-NNDS. Data are expressed as mean ± standard deviation. Reported *p* values are for between-group comparisons (Kruskal–Wallis test). False discovery rate was controlled at 5% by the Benjamini–Hochberg method. Significant differences were followed up by post-hoc paired comparisons (Dunn–Sidak test). Abbreviations: MNA, mini Nutritional assessment; TAAs, total amino acids; BCAAs, branched chain amino acids; EAAs, essential amino acids.

**Table 3:** Levels of plasma amino acids (µmol/L) and statistical analysis between combined group (CG = malnourished-MNA Group 1 + at risk of malnutrition-MNA Group 2) and normonourished-MNA Group 3 patients according to Mini Nutritional Assessment (MNA).

Amino Acids	CG (*N* = 26)	MNA Group 3 (*N* = 28)	*p* Value
Aspartic acid	7.69 ± 3.83	8.86 ± 6.80	0.63
Glutamic acid	61.65 ± 17.64	68.68 ± 17.70	0.09
Histidine	67.92 ± 11.99	71.39 ± 14.14	0.16
Asparagine	39.62 ± 5.87	42.71 ± 6.51	0.10
Serine	98.27 ± 21.51	110.00 ± 48.16	0.39
Glutamine	504.42 ± 81.73	511.07 ± 82.65	0.67
3-methyl-histidine	3.53 ± 1.95	3.88 ± 1.30	0.37
Arginine	66.31 ± 22.10	63.79 ± 22.33	0.59
Citrulline	34.19 ± 9.09	35.32 ± 11.75	0.94
Glycine	242.65 ± 80.24	232.79 ± 81.78	0.47
Threonine	119.42 ± 26.99	118.07 ± 28.58	0.56
Alanine	356.23 ± 81.66	344.14 ± 74.21	0.64
Taurine	74.96 ± 43.53	64.39 ± 15.10	0.50
Tyrosine	53.85 ± 12.85	53.21 ± 12.47	0.92
Tryptophan	43.58 ± 10.72	46.11 ± 8.36	0.25
Phenylalanine	54.88 ± 15.89	53.11 ± 10.19	0.73
Isoleucine	55.15 ± 18.60	60.00 ± 14.70	0.30
Leucine	103.23 ± 31.54	116.79 ± 24.39	0.047
Lysine	202.58 ± 49.50	205.21 ± 28.65	0.48
Valine	201.30 ± 61.51	227.73 ± 47.55	0.047
TAAs	2400.22 ± 400.28	2446.96 ± 308.65	0.72
BCAAs	359.68 ± 110.39	404.52 ± 84.87	0.060
EAAs	851.60 ± 202.24	902.29 ± 145.03	0.17
EAAs/TAAs	0.35 ± 0.04	0.37 ± 0.02	0.023 ^^^
BCAAs/TAAs	0.15 ± 0.03	0.16 ± 0.02	0.009 ^^^
BCAAs/EAAs	0.42 ± 0.04	0.45 ± 0.03	0.002 ^^^

Data are expressed as mean values ± standard deviation. Reported *p* values are for between group comparisons (Kruskal–Wallis test). Only *p* values followed by the symbol ^^^ remained significant after control for false discovery rate. Abbreviations: MNA, mini nutritional assessment; TAAs, total amino acids; BCAAs, branched chain amino acids; EAAs, essential amino acids.

**Table 4:** Non-amino acid variables of the population of patients with Alzheimer’s disease (AD) subdivided into malnourished patients (MNA Group 1), patients at risk of malnutrition (MNA Group 2), normonourished patients (MNA Group 3), and combined group (CG = MNA Group 1 + MNA Group 2).

Variable	CG (*N* = 26)	MNA Grp 1 (*N* = 8)	MNA Grp 2 (*N* = 18)	MNA Grp 3 (*N* = 28)	*p* Value CG vs. Grp 3
**Demographic variables**					
Age (years)	74.58 ± 9.01	75.38 ± 5.42	74.22 ± 10.33	74.32 ± 7.11	0.81
Male gender (%)	17 (65%)	5 (62%)	12 (67%)	20 (71%)	0.07
**Anthropometric variables**					
Education (years)	7.11 ± 4.08	8.33 ± 4.59	6.54 ± 3.89	7.41 ± 3.75	0.84
Body weight (kg)	59.15 ± 11.46	55.70 ± 13.42	60.68 ± 10.52	68.11 ± 12.83	0.006
Height (cm)	158.42 ± 10.82	162.00 ± 8.64	156.83 ± 11.52	163.14 ± 8.66	0.10
Body mass index (kg/m^2^)	23.76 ± 5.00	21.58 ± 6.52	24.73 ± 4.00	25.60 ± 4.49	0.09
Mid-arm circumference (cm)	25.56 ± 3.24	23.44 ± 3.83	26.50 ± 2.53	26.43 ± 2.36	0.34
**Biohumoral variables**					
Glucose (NV 70–115 mg/dL)	85.50 ± 9.74	86.00 ± 5.93	85.28 ± 11.18	90.36 ± 14.56	0.36
Insulin (NV 4–24 µU/mL)	11.10 ± 4.37	9.89 ± 3.37	11.64 ± 4.73	13.52 ± 5.53	0.11
HOMA-IR (NV < 2.4)	3.41 ± 1.99	2.55 ± 1.07	3.79 ± 2.21	4.07 ± 1.89	0.15
Glycosylated haemoglobin (4.8–5.9%)	5.53 ± 0.51	5.58 ± 0.21	5.52 ± 0.60	5.61 ± 0.88	0.69
Total cholesterol (NV < 200 mg/dL)	188.77 ± 32.44	196.50 ± 36.26	185.33 ± 31.08	186.04 ± 32.87	0.96
HDL cholesterol (NV: M > 55 mg/dL; F > 65 mg/dL)	56.85 ± 17.72	52.88 ± 12.53	58.61 ± 19.66	53.68 ± 14.05	0.71
LDL cholesterol (NV < 100 mg/dL)	111.69 ± 25.28	123.25 ± 23.43	106.56 ± 24.96	110.25 ± 26.96	0.89
Transferrin (NV 200–360 mg/dL)	230.69 ± 33.89	220.25 ± 40.08	235.33 ± 30.88	229.07 ± 41.89	0.72
Iron (NV 59–158 µg/dL)	86.38 ± 29.37	91.13 ± 26.63	84.28 ± 31.01	83.43 ± 22.24	0.72
Tryglycerides (NV 0–200 mg/dL)	99.00 ± 34.58	100.13 ± 45.82	98.50 ± 29.89	105.36 ± 43.55	0.94
Vitamin B12 (NV 191–663 pg/mL)	331.96 ± 139.31	446.88 ± 150.31	277.88 ± 97.56	318.00 ± 162.25	0.47
Folate (NV 3.1–17.5 ng/mL)	6.47 ± 3.49	6.21 ± 3.66	6.59 ± 3.52	6.64 ± 2.93	0.49
Creatinine (NV: M 0.73–1.18 mg/dL; F 0.55–1.02 mg/dL)	0.90 ± 0.24	0.97 ± 0.33	0.86 ± 0.18	0.91 ± 0.21	0.66
Total proteins (NV 6.6–8.7 g/dL)	6.33 ± 0.49	6.29 ± 0.79	6.35 ± 0.30	6.27 ± 0.33	0.79
Albumin (NV 3500–5200 mg/dL)	3683.85 ± 360.31	3572.50 ± 560.91	3733.33 ± 230.09	3728.93 ± 284.84	0.54
White blood cell count (NV 4.00–10.00 × 10^3^/µL)	6.59 ± 1.79	7.16 ± 2.12	6.32 ± 1.61	6.11 ± 1.21	0.60
Red blood cell count (NV: M 4.30–5.70 × 10^6^/µL; F 3.80–5.20 × 10^6^/µL)	4.20 ± 0.46	4.21 ± 0.41	4.19 ± 0.50	4.47 ± 0.44	0.025
Haemoglobin (NV: M 13.2–17.3 g/dL; F 11.7–15.5 g/dL)	12.81 ± 1.31	12.70 ± 0.95	12.86 ± 1.47	13.44 ± 1.18	0.035
Erythrosedimentation rate (NV < 15 mm/1st h)	23.28 ± 22.19	24.25 ± 24.67	22.82 ± 21.71	13.00 ± 12.16	0.07
**AD biomarkers in CSF**					
tau (NV < 404 pg/mL)	486.86 ± 609.36	231.60 ± 107.46	628.67 ± 730.94	444.14 ± 216.02	0.14
p-tau (NV < 56.5 pg/mL)	72.07 ± 44.15	45.60 ± 20.13	86.78 ± 47.78	79.20 ± 28.62	0.28
β-amyloid (NV > 599 pg/mL)	532.29 ± 471.59	343.60 ± 172.28	637.11 ± 558.52	566.95 ± 283.63	0.18
β-amyloid/tau (NV > 1.6)	2.46 ± 3.43	1.94 ± 1.35	2.74 ± 4.23	2.02 ± 2.31	0.96
β-amyloid/p-tau (NV > 11.5)	9.92 ± 10.33	9.52 ± 7.52	10.14 ± 12.04	8.33 ± 6.32	0.99
**Neurocognitive tests**					
Mini Mental State Examination (MMSE < 24 denotes cognitive impairment)	15.75 ± 7.30	17.17 ± 5.75	15.11 ± 8.07	19.14 ± 7.13	0.20
Digit span (NV < 3.75)	4.06 ± 0.77	4.92 ± 0.52	3.63 ± 0.41	3.78 ± 1.00	0.52
Trail Making Test TMT-A (NV < 93″)	162.00 ± 56.19	178.00 ± 33.94	155.60 ± 65.34	145.21 ± 55.31	0.65
Trail Making Test TMT-B (NV < 282″)	262.00 ± 136.96	347.00 ± 0.00	219.50 ± 163.34	162.33 ± 94.98	0.24

Data are expressed as mean ± standard deviation except for gender, expressed as *N* (%). Reported *p* values are for between-group comparison (CG vs. MNA Group 3, Kruskal–Wallis test). Abbreviations: MNA, Mini Nutritional Assessment; NV, normal values; HOMA-IR, HOmeostasis Model Assessment-Insulin Resistance; CSF, cerebrospinal fluid.
